# Virtual Humans in Virtual Reality Mental Health Research: Systematic Review

**DOI:** 10.2196/75087

**Published:** 2025-10-08

**Authors:** Shu Wei, Daniel Freeman, Aitor Rovira

**Affiliations:** 1 Department of Psychiatry, University of Oxford, Warneford Hospital, Warneford Ln, HeadingtonOxford, OX3 7JX, United Kingdom; 2 Department of Paediatrics, Yale School of Medicine, New Haven, CT, United States; 3 Department of Experimental Psychology, University of Oxford, Oxford, United Kingdom; 4 Oxford Health NHS Foundation Trust, Oxford, United Kingdom

**Keywords:** virtual reality, virtual human, agent, avatar, mental health

## Abstract

**Background:**

Virtual reality (VR) is showing increasing promise for assessing, understanding, and treating mental health difficulties. Virtual humans (VHs) represent a key aspect within many VR mental health applications. While VHs can play diverse roles and display varied characteristics, their design and influence have rarely been the primary focus of mental health research.

**Objective:**

We aimed to carry out a systematic review of how VHs in immersive VR have been used in applications for mental health, focusing on their roles and interaction types, and the human characteristics being tested.

**Methods:**

Following the PRISMA (Preferred Reporting Items for Systematic Reviews and Meta-Analyses) guidelines, we searched PubMed, MEDLINE, PsycINFO, Scopus, and Web of Science, using defined keyword combinations involving VR, VHs, and mental health. Eligible studies included peer-reviewed research using immersive VR with VHs in a mental health context, without restrictions on study design or population. We excluded nonimmersive VR, nonmental health applications, and papers without empirical data. Data were synthesized narratively, and a taxonomy to categorize VHs that we developed was used.

**Results:**

A total of 79 studies met all eligibility criteria. VHs were most frequently applied in studies on social anxiety (n=18), eating disorders (n=18), and psychosis (n=15). They were primarily used as active social interaction partners (n=40), as part of virtual crowds (n=16), and as virtual bodies for participants (n=23). Explicit interactions dominated active partner studies, while implicit and passive or no interactions were prevalent in crowd and body studies. Over half of the studies (n=44) varied the VH characteristics, with body size and gender being the most common variables, and personality was explored in fewer studies (n=5). Only a limited number of studies provided detailed descriptions of VH appearance and behavior, with some including still images and videos.

**Conclusions:**

VHs are versatile tools to be used within VR mental health applications, but their design features are inconsistently reported and insufficiently examined in relation to intervention outcomes. Evidence is limited by heterogeneity in study aims, designs, and populations, and by incomplete reporting of VH characteristics, which constrains replication and cross-study comparison. Standardized reporting and systematic investigations of VH design are needed to optimize their roles in evidence-based mental health applications.

## Introduction

Virtual humans (VHs) have been part of virtual reality (VR) mental health applications since the late 1990s. The pioneering work by Riva and Melis [1] used images of a female silhouette in VR to represent the participant’s body, marking one of the earliest uses of VHs in mental health research. Other early studies showed that virtual audiences could effectively induce anxiety in participants [2,3]. Since approximately 2013, advancements in VR technology and reductions in cost have significantly improved accessibility, enabling the use of VHs in research areas such as understanding paranoia [4,5], managing autism-centered interventions [6,7], and improving symptoms from posttraumatic stress disorder (PTSD) [8]. Some recent applications have integrated virtual coaches to guide patients and automate treatments [9,10], as well as body swapping experiences that allow participants to switch between different entities to experience perspective changes [11-13].

VHs enable researchers to simulate social interactions commonly encountered in daily life [14,15] or under special circumstances such as conflict or danger [16,17]. They can also serve as the self-representations of participants through the sense of embodiment and the creation of the illusion that the virtual body they see and control is their own body [18]. Compared to experimental setups with trained real actors, VHs provide greater experimental control and repeatability, preserving ecological validity [19].

Despite their growing use and potential, VHs have not been studied thoroughly in mental health research. This gap is partly due to the inherently multidisciplinary nature of the field, which requires expertise in psychology, immersive technologies, and 3D design to build VHs that are clinically suitable and technically feasible. Furthermore, conducting full-scale clinical trials to evaluate VH design within patient populations is often impractical. In contrast, the computer science community has explored VHs to optimize their visual and animation quality [20-22], develop realistic and adaptive behaviors [23-25], and create open-source character libraries [26-28]. These technical advances offer promising opportunities to extend the applications of VHs in mental health beyond their current use.

Understanding the role and impact of VHs in mental health research is essential for optimizing current implementations and discovering future applications. As VR and character creation software tools improve, VHs have the potential to play an even more important role in mental health, offering customized therapeutic experiences with fine-tuned emotional attributes designed specifically for each patient. We present a systematic review of research studies in mental health VR featuring VHs, from the early development of VR until November 2024. This review proposes a taxonomy based on the most important aspects to consider in VHs for mental health applications and classifies the studies found in this domain. The systematic review addresses the following questions:

- What roles and functions have VHs played in VR mental health research?
- What have been the chosen agencies for VHs in VR mental health research?
- What characteristics of VHs have been examined in VR mental health research?

## Methods

### Categorizing VHs in Mental Health VR

**Figure 1.**
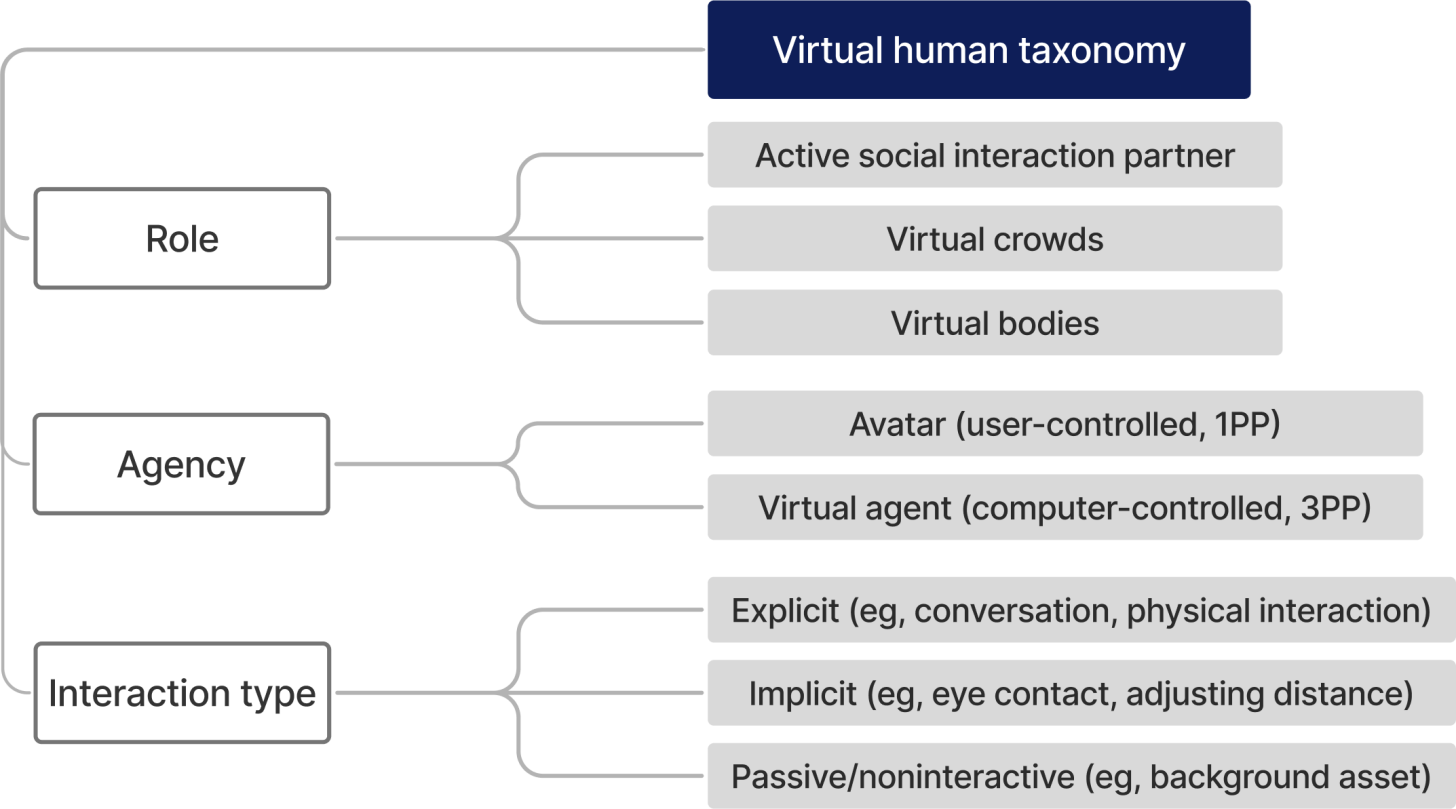
A taxonomy of virtual humans based on existing mental health virtual reality studies. 1PP: first-person perspective; 3PP: third-person perspective.

We present a taxonomy of VHs in mental health VR (Figure 1), with categorization of their features into 3 main aspects: role, agency, and interaction type. These features were selected because they represent the critical dimensions influencing how VHs are designed, implemented, and experienced in mental health VR. Role defines the primary function of the VH within the virtual environment, agency determines the level of control and autonomy the VH exhibits, and interaction type describes the nature and depth of engagement between the VH and the participant. Together, these features provide a framework for understanding the diverse ways VHs are used in mental health research, enabling researchers to systematically evaluate their design and impact.

This framework was inductively derived from the included studies, and it was also supported by prior classification approaches in VR and virtual character research. Existing taxonomies highlight comparable dimensions—distinguishing characters according to their functional role [29,30], level of autonomy or agency [19,29,31,32], and communicative capacity or interaction features [19,30]. By aligning with these established design variables while adapting them to the specific context of mental health VR, our taxonomy provides both empirical grounding and theoretical consistency with prior frameworks.

#### Role

The role of VHs can be divided into 3 categories: active social interaction partners, virtual crowds, and virtual bodies. These categories were chosen to reflect the distinct ways VHs are used in VR mental health research across diverse applications. They emerged from recurring functional patterns observed across studies and align with prior literature around VHs, which supports the distinction among interactive agents, crowds, and avatars as the most common roles [33-35]. For example, Kyrlitsias and Michael-Grigoriou [33] differentiated VHs by their interaction scenario and level of engagement, while other researchers have examined the psychological impacts of virtual crowds compared to individual characters [34,36]*.*

Active social interaction partners engage with participants to simulate interactions through verbal and nonverbal communication [13,37]. A virtual coach is an example of an active interaction partner that focuses on providing guidance or feedback to facilitate psychological treatment [9]. Virtual crowds, unlike active social interaction partners, have limited contact with participants or normally interact passively with implicit interaction, such as making eye contact [38]. It is common for virtual crowds to be part of the stimuli in the virtual world [39]. Virtual bodies focus on body representation, either by depicting a range of body types [1] or serving as the user’s avatar for embodiment experiences [40].

#### Agency Avatars and Agents

Agency refers to the state or entity of taking action or exerting control [41] and can be categorized as avatars and virtual agents (VAs).

An avatar is a digital representation controlled by a user, serving as their own body in a different medium, for example, in a virtual environment [41]. Typically viewed from a first-person perspective, avatars are co-located with the user’s real body. The real body is usually concealed, thus overriding them and creating the illusion that the body they see is their own body. The term “avatar” has often been misused in VR studies to describe any VH, even those who do not represent any user and are controlled by a computer.

A VA is a computer-generated character designed to simulate human interaction autonomously, moving and responding without being controlled by a real person [42]. VAs are mainly controlled by computers and typically viewed from a third-person perspective. They can exhibit varying degrees of autonomy, with semiautonomous agents combining computer automation with operator intervention. This approach is referred to as the Wizard of Oz technique [43] (a hidden operator simulates autonomous system behavior) and allows researchers to make VAs respond in real-time based on participant responses while still being computer-animated [19].

#### Interaction Types of VAs

VAs can have different degrees of interaction with human participants. We have categorized them into explicit, implicit, and passive or noninteractive.

Explicit interactions involve direct engagement between a VA and a participant, such as conversations, physical interactions (eg, shaking hands [44]), or collaborative activities (eg, playing a sport together [45]). Explicit interactions aim to replicate real-life social experiences, and they can be difficult to implement due to the complexity of managing a large pool of possible outcomes.

Implicit interactions include subtle behaviors, such as acknowledging the participant’s presence by making eye contact or adjusting interpersonal distance, without fully engaging in an explicit interaction. Such interactions are less resource-intensive than explicit ones and still contribute to enhancing the sense of presence [46,47], making participants feel as if they are part of the scene rather than just being spectators.

Passive or noninteractive VAs act as background elements in VR environments, unresponsive to participant actions. They are easier to implement due to the scripted nature of their animations. They are used for crowd simulations and creating social situations such as populating a virtual restaurant [48] or constructing an office scene [49].

### Inclusion and Exclusion Criteria

The inclusion and exclusion criteria for eligible studies are summarized in Textbox 1. In brief, studies were required to (1) use VHs within an immersive VR setting; (2) involve assessment or intervention related to mental health conditions; and (3) include empirical data from human participants. Studies were excluded if they were not related to mental health, did not involve a VR-based VH, or were not available as peer-reviewed full-text articles in English.

Textbox 1. **Inclusion and exclusion criteria.**

**Inclusion criteria**

- Population: Human participants
- Technology use: Immersive virtual reality (VR) with virtual human (VH) characters
- Application area: Mental health assessment or intervention
- Study type: Empirical study involving more than one participant with data collection
- Other criteria: English; available as full text

**Exclusion criteria**

- Population: Nonhuman studies
- Technology use: VR without VH or nonimmersive VR (eg, desktop system)
- Application area: Not related to mental health
- Study type: Reviews, protocols, abstracts only, commentaries, and opinion pieces
- Other criteria: Non-English; conference abstract

### Search Strategy

The literature search was performed in PubMed, Scopus, MEDLINE (Ovid), PsycINFO (Ovid), and Web of Science (Clarivate), with title and abstract searches for keyword combinations involving (1) VR, (2) VH, and (3) mental health. The keyword query was structured as follows: [(virtual reality OR immersive virtual reality OR VR) AND (virtual human OR virtual character OR virtual agent OR avatar OR humanoid) AND ((assessment OR treatment OR therapy OR mental health) OR (mood disorders OR depress* OR bipolar OR mania OR paranoia OR psychosis OR psychotic OR schizophren* OR schizotyp* OR delus* OR hallucinat* OR phobias OR obsessive compulsive disorder OR OCD OR anxiety OR post traumatic stress disorder OR PTSD OR trauma OR anorexia nervosa OR bulimia nervosa OR eating disorders OR binge eating OR insomnia OR sleep OR nightmares OR circadian OR panic OR substance OR abuse OR cannabis OR tobacco OR alcohol OR amphetamine OR hallucinogens OR heroin))]. Details of the searches for each database in the corresponding platforms can be found in Multimedia Appendix 1. A completed PRISMA (Preferred Reporting Items for Systematic Reviews and Meta-Analyses) checklist [50] is presented in Checklist 1.

### Article Screening

Following database searches, we identified 2596 records (Scopus, n=1232; Web of Science, n=712; PubMed, n=233; APA PsycINFO, n=247; MEDLINE, n=172) between 1997 and 2024 (final search: November 20, 2024). After removing 1085 duplicates (11 manually and 1074 via Covidence), 1511 unique records remained. Title and abstract screening excluded 1280 records that did not involve a VH character, did not use immersive VR, were unrelated to mental health, were nonhuman studies, or were non-English studies. The remaining 231 full-text articles were then assessed in detail, with the reasons for exclusion including the following: not immersive VR (n=73), not related to mental health (n=31), not full paper (eg, conference abstract) (n=21), no empirical data (n=19), no VH (n=11), and case study with only 1 participant (n=3).

## Results

### Search Results

**Figure 2.**
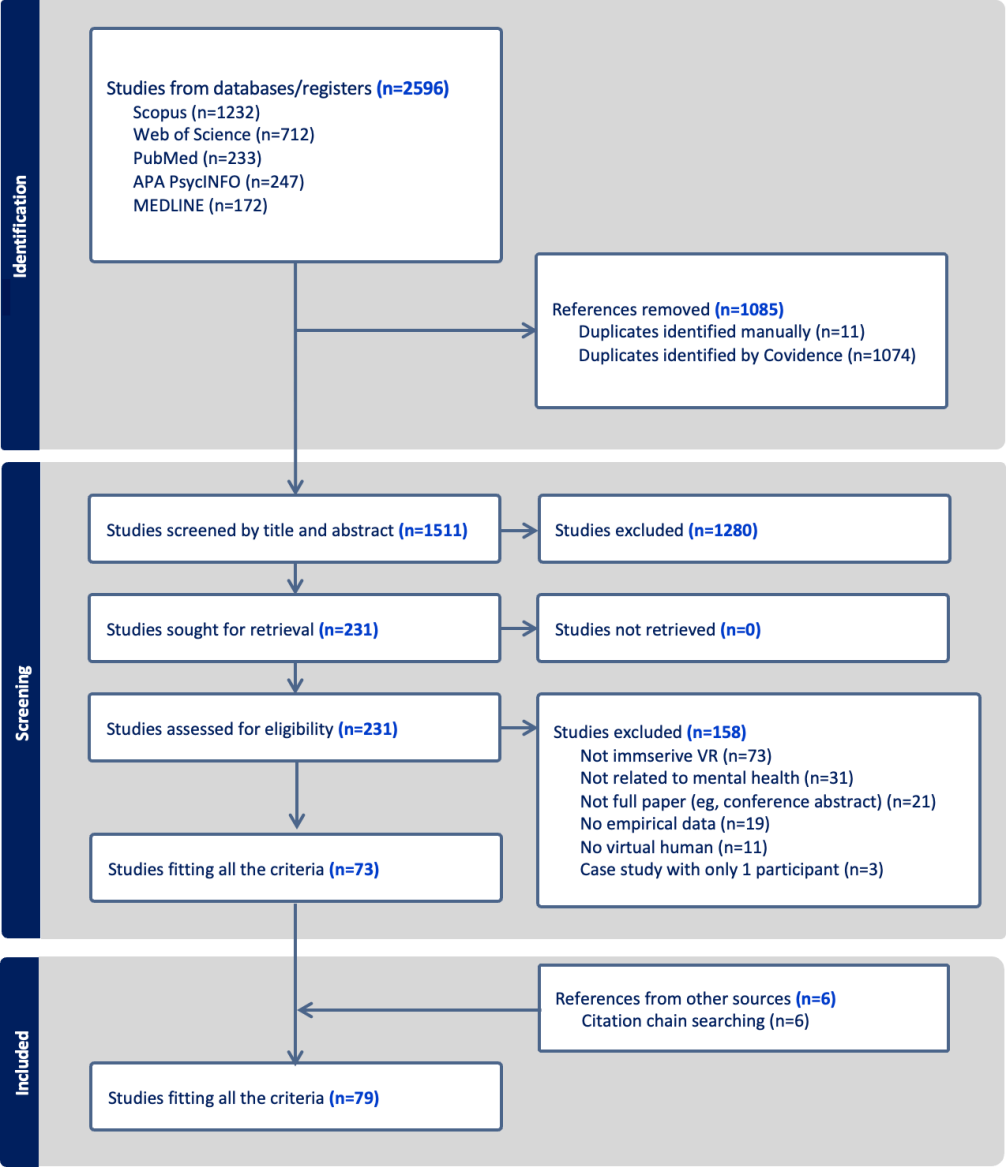
PRISMA (Preferred Reporting Items for Systematic Reviews and Meta-Analyses) flow diagram. VR: virtual reality.

Figure 2 (PRISMA diagram [50]) summarizes the search and screening results. In total, 79 studies were included in the review: 73 that met all eligibility criteria at full-text screening and 6 identified through citation chaining. We have provided a list of studies that primarily used VHs as active social interaction partners (Table 1), virtual crowds (Table 2), and virtual bodies (Table 3), along with a brief summary for each.

**Table 1.** Studies that primarily used VHs^a^ as active social interaction partners.

Study	Role	Agency	Virtual agent interaction type	Parameters for manipulation	Condition	Participants, n	Participant characteristics	Comments on findings
Lee et al [51], 2004	Active interaction partner	Agent	Implicit interaction	—^b^	Substance abuse	16	Males who smoked at least 10 cigarettes a day	VHs in cue exposure therapy exerted a stronger impact compared to seductive objects.
Park et al [52], 2009	Active interaction partner	Agent	Explicit interaction	Gender; Emotional facial expression	Psychosis	54	Patients diagnosed with schizophrenia and the nonclinical general population	VHs with emotional facial expressions successfully triggered emotional and biological responses.
Wieser et al [53], 2010	Active interaction partner	Agent	Implicit interaction	Gender; Gaze pattern; Interpersonal distance	Social anxiety	39	Females with social anxiety	Women paid more attention to the weight-gain areas on VHs than men.
Kwon et al [54], 2013	Active interaction partner	Agent	Explicit interaction	—	Social anxiety	20	Students with social anxiety	VHs provoked both stronger anxiety and presence than those in a nonimmersive display.
Powers et al [55], 2013	Active interaction partner	Semiautonomous agent	Explicit interaction	—	Social anxiety	26	Female undergraduates	Encountering VHs could trigger strong anxiety and fear.
Han et al [56], 2014	Active interaction partner	Agent	Explicit interaction	Attitude (positive, negative dialog)	Psychosis	45	Patients with schizophrenia and the nonclinical general population	Patients with schizophrenia had active avoidance of eye contact with VHs compared to healthy controls.
Hartanto et al [57], 2014	Active interaction partner	Semiautonomous agent	Explicit interaction	Attitude (positive, negative dialog)	Social anxiety	24	General population	Positive dialog feedback from VHs resulted in less anxiety, lower heart rate, and longer answers.
Pan et al [58], 2015	Active interaction partner	Semiautonomous agent	Explicit interaction	Personality (confident, shy)	Social anxiety	24	Male participants	VHs with a shy personality can be perceived as more friendly.
Fornells-Ambrojo et al [4], 2016	Active interaction partner	Semiautonomous agent	Explicit interaction	Responsiveness in social interaction	Psychosis	61	Male participants	Highly contingent VHs were perceived as more trustworthy for extremely paranoid individuals.
Ryan and Griffin [59], 2016	Active interaction partner	Agent	Implicit interaction	—	Social anxiety	27	Students	VR^c^ exposure to a VH could successfully trigger social anxiety.
Falconer et al [11], 2016	Active interaction partner	Agent and/or avatar	Explicit interaction	—	Depression	15	Patients with depression	Changing the viewer perspective in a virtual social interaction could decrease self-criticism and increase self-compassion.
Robitaille et al [60], 2017	Active interaction partner	Agent and/or avatar	Explicit interaction	Responsiveness in social interaction	PTSD^d^	12	Nonclinical military members and military members with mild traumatic brain injury	Participants with PTSD displayed a lack of navigational behavior during a VH interaction.
Amaral et al [6], 2018	Active interaction partner	Agent	Implicit interaction	—	Autism	15	Patients with high-functioning ASD^e^	VH affected social attention among patients with autism.
Percie du Sert et al [61], 2018	Active interaction partner	Semiautonomous agent	Explicit interaction	—	Psychosis	15	Patients diagnosed with schizophrenia	VR therapy with VH could improve auditory verbal hallucinations and depressive symptoms.
Freeman et al [62], 2018	Active interaction partner (virtual coach)	Agent	Explicit interaction	—	Phobias	100	Individuals with a fear of heights	A virtual therapist could help automate the VR therapy.
Shin et al [63], 2018	Active interaction partner	Agent	Explicit interaction	Different levels of social pressure	Others	64	General population and patients with internet gaming disorder	Exposure to VHs in gaming cafes triggered gaming cravings.
Kothgassner et al [64], 2019	Active interaction partner	Agent and/or avatar	Explicit interaction	Perceived agency (avatar vs agent)	Social anxiety	56	Students	Virtual social support from a VH could be effective when the recipient thought it was from a human.
Quintana et al [65], 2019	Active interaction partner	Agent	Explicit interaction	—	Social anxiety	53	Female adults	Perception toward the VH could be affected by odorant manipulation.
Reichenberger et al [66], 2019	Active interaction partner	Agent	Explicit interaction	Gender; Interaction pattern (with or without aversive behavior)	Social anxiety	60	General population	VHs could be used to learn about social fear, and women reported higher fear compared to men.
Slater et al [13], 2019	Active interaction partner (virtual coach)	Agent and/or avatar	Explicit interaction	Dialog contents (self-dialog versus scripted dialog)	Depression	58	General population	Self-dialog VR coaching had better results than scripted VR counseling.
Seo et al [67], 2019	Active interaction partner	Agent	Explicit interaction	Interaction pattern (combination of gaze and pointing)	Social anxiety	33	General population	VHs with nonverbal behavior could be used to measure and promote joint attention.
Miloff et al [68], 2020	Active interaction partner (virtual coach)	Agent	Explicit interaction	—	Phobias	70	Patients with a fear of spiders	Alliance toward a virtual therapist is a significant predictor of treatment outcome in VR.
Nijman et al [69], 2020	Active interaction partner	Semiautonomous agent	Explicit interaction	Facial expression	Psychosis	22	Patients with psychotic disorders	VHs’ facial expressions can be used to train participants’ social cognition.
Kim et al [70], 2020	Active interaction partner	Agent	Explicit interaction	—	Others	36	Nonclinical male volunteers	VHs could be used for assessment and intervention to promote subjective well-being.
Lee et al [71], 2021	Active interaction partner	Agent and/or avatar	Explicit interaction	—	Psychosis	48	Patients diagnosed with schizophrenia and the nonclinical general population	The social context created by VHs impacted the difference in peripersonal space recognition.
Kothgassner et al [45], 2021	Active interaction partner	Agent and/or avatar	Explicit interaction	Perceived agency (avatar vs agent)	Social anxiety	84	Females	Social interaction experience with VHs was comparable to the real-life experience of cyberbullying.
Brander et al [72], 2021	Active interaction partner	Semiautonomous agent	Explicit interaction	Visual and behavioral traits of the virtual character through customization	Psychosis	109	Psychiatric hospital staff (psychotherapists, nursing staff, and administrators)	The function to customize VHs for VR treatment was highly valued by clinical staff.
Guldager et al [73], 2022	Active interaction partner	Agent	Explicit interaction	—	Substance abuse	372	Students aged 15‐18 years	VR party simulation could improve adolescents’ drinking refusal skills.
Freeman et al [9], 2022	Active interaction partner (virtual coach)	Agent	Explicit interaction	—	Psychosis	346	Patients with psychosis	Automated VR therapy with VHs could reduce anxious avoidance and distress in everyday situations.
Fusaro et al [7], 2023	Active interaction partner	Agent and/or avatar	Explicit interaction	—	Autism	53	General population and individuals with ASD	Autistic adults exhibited greater interpersonal distance with VHs.
Bektas et al [74], 2023	Active interaction partner	Agent	Explicit interaction	—	Eating disorder	70	Patients with anorexia nervosa	The addition of a VH to the virtual kitchen scene could increase the association between food-specific state disgust and symptoms of eating disorders.
Giguère et al [75], 2023	Active interaction partner	Semiautonomous agent	Explicit interaction	Voice and facial expressions	Substance abuse	19	Patients with cannabis use disorder and severe mental disorders	The VH intervention could reduce cannabis use among people with cannabis use disorder and severe mental disorders.
Halim et al [12], 2023	Active interaction partner	Agent and/or avatar	Explicit interaction	—	Depression	36	Young adults	Changing the viewer perspective through a virtual body swapping experience can help enhance self-compassion and reduce depressive symptoms.
Wei et al [37], 2023	Active interaction partner (virtual coach)	Agent	Explicit interaction	Head nods and facial expressions	Phobias	120	General population with a fear of heights	The detailed design of a virtual coach’s emotional attributes could affect people’s therapeutic alliance and their confidence in the VR therapy.
Artiran et al [76], 2024	Active interaction partner	Agent	Explicit interaction	Levels of attention (degrees of backchannels)	Autism	30	Patients with autism and the nonclinical population	The behavior of virtual interviewers influenced the gazes and head movements in individuals with autism.
Natali et al [77], 2024	Active interaction partner	Agent	Explicit interaction	—	Eating disorder	145	Patients with anorexia nervosa	VHs could provide positive social support to patients with anorexia nervosa in a VR food exposure scene.
Hidding et al [78], 2024	Active interaction partner	Agent	Explicit interaction	—	Others	68	Undergraduate students with high levels of self-criticism	Expressing compassion to a VH with similar self-criticism could reduce self-criticism and increase self-compassion.
Freeman et al [10], 2024	Active interaction partner (virtual coach)	Agent	Explicit interaction	—	Psychosis	11	Patients with nonaffective psychosis and low positive self-beliefs (aged 16‐26 years)	Automated VR therapy with a virtual coach could improve self-beliefs and well-being in young patients with psychosis.
Yamashita and Yamamoto [79], 2024	Active interaction partner (virtual coach)	Agent and/or avatar	Explicit interaction	Identity of the VH (Freud or an intimate person of the participant)	Social anxiety	60	Students with personal problems	VR self-counseling with the intimate other avatar was most effective in improving people’s anxiety.
Banakou et al [80], 2024	Active interaction partner	Agent	Explicit interaction	—	Social anxiety	45	General population with public speaking anxiety	Single-session VR exposure therapy with gradually increasing stimulus intensity may be as effective as multisession exposure for public speaking anxiety.

a VH: virtual human.

b Not applicable.

c VR: virtual reality.

d PTSD: posttraumatic stress disorder.

e ASD: autism spectrum disorder.

**Table 2.** Studies that primarily used VHs^a^ as virtual crowds.

Study	Role	Agency	Virtual agent interaction type	Parameters for manipulation	Condition	Participants, n	Participant characteristics	Comments on findings
Slater et al [3], 1999	Virtual crowds	Semiautonomous agent	Implicit interaction	Attentional focus level	Social anxiety	10	General population	A virtual audience could be used to treat public speaking fears; Higher perceived audience interest reduced public speaking anxiety.
Tarnanas et al [49], 2003	Virtual crowds	Agent	Passive interaction	—^b^	Social anxiety	120	General population and patients with work-related stress disorders	Virtual crowds triggered psychological and biological reactions in social anxiety.
Freeman et al [81], 2003	Virtual crowds	Agent	Passive interaction	—	Psychosis	24	General population	People tend to attribute their mental states to VHs.
Freeman et al [82], 2005	Virtual crowds	Agent	Passive interaction	—	Psychosis	30	People covering the whole range of paranoia	Neutral characters could elicit people’s persecutory thoughts and be used to understand psychosis.
Freeman et al [15], 2008	Virtual crowds	Agent	Passive interaction	—	Psychosis	200	General population	Neutral characters could elicit paranoid ideation among the general population.
Cho et al [83], 2008	Virtual crowds	Agent	Implicit interaction	—	Substance abuse	10	Population with a low level of alcohol dependence	The presence of a VH in cue exposure induced stronger cravings.
Rizzo et al [39], 2010	Virtual crowds	Agent	Implicit interaction	—	PTSD^c^	20	Active duty service members	A VR^d^ environment with VHs could simulate a war scene for the clinical treatment of PTSD and depression.
Brinkman et al [48], 2011	Virtual crowds	Agent	Passive interaction	Density; Ethnic appearance (White European or North African)	Social anxiety	26	General population and patients diagnosed with schizophrenia	VR exposure to an increased density and proportion of VHs with other ethnicities induced strong physiological arousal and distress.
Shiban et al [84], 2015	Virtual crowds	Agent	Passive interaction	—	Social anxiety	40	General population	VHs can be used to understand social fears in VR.
Mountford et al [85], 2016	Virtual crowds	Agent	Passive interaction	—	Eating disorder	18	General population	The exposure to VHs did not necessarily change people’s in-state body image.
Atherton et al [86], 2016	Virtual crowds	Agent	Passive interaction	—	Psychosis	26	Males reporting paranoid ideation within the past month	People’s own self-confidence could affect their paranoid thoughts toward the VHs.
Gürerk et al [14], 2019	Virtual crowds	Agent	Passive interaction	Different working behaviors	Social anxiety	108	General population	VHs with better working performance could motivate people to perform better.
Takac et al [87], 2019	Virtual crowds	Agent	Implicit interaction	Crowd size; Attentional focus level	Social anxiety	19	General population	Virtual audiences could elicit public speaking distress, which could last beyond VR sessions.
Wei et al [88], 2024	Virtual crowds	Agent	Implicit interaction	Facial expressions	Psychosis	122	General population with elevated paranoia	The detailed design of VH faces affected people’s paranoid interpretations.
Gayer-Anderson et al [89], 2025	Virtual crowds	Agent	Implicit interaction	—	Psychosis	481	Adolescents	VR school scenes with VHs could help understand the precursors of adolescents’ paranoid ideation.
Rovira et al [90], 2024	Virtual crowds	Agent	Explicit interaction	—	Substance abuse	100	People who smoke daily	VR exposure to smoking-cue VHs elicited stronger cigarette cravings than a neutral environment without VHs.

a VH: virtual human.

b Not applicable.

c PTSD: posttraumatic stress disorder.

d VR: virtual reality.

**Table 3.** Studies that primarily used VHs^a^ as virtual bodies.

Study	Role	Agency	Virtual agent interaction type	Parameters for manipulation	Condition	Participants, n	Participant characteristics	Comments on findings
Riva and Melis [1], 1997	Virtual body	Agent	Passive interaction	Body size	Eating disorder	119	General population	Virtual figures could be used to understand people’s body images.
Perpiñá et al [91], 1999	Virtual body	Agent	Passive interaction	Body size	Eating disorder	13	Patients with eating disorders	VR^b^ body image treatment could have a stronger impact than standard treatment.
Aymerich-Franch et al [92], 2014	Virtual body	Avatar	Passive interaction	Different levels of visual similarity to the participant	Social anxiety	187	General population	Having an avatar with an insimilar face could reduce anxiety.
Ferrer-Garcia et al [40], 2017	Virtual body	Agent	Passive interaction	Body size	Eating disorder	23	College students	Owning a fatter virtual body triggered higher levels of body anxiety and fears of weight gain.
Ferrer-Garcia et al [93], 2018	Virtual body	Agent	Passive interaction	Body size	Eating disorder	40	Female students	Owning a virtual body with different body sizes produced changes in body dissatisfaction.
Porras-Garcia et al [94], 2018	Virtual body	Agent	Passive interaction	Body size	Eating disorder	35	College students	Owning a larger-size virtual body increased people’s body checking behaviors.
Porras-Garcia et al [95], 2019	Virtual body	Agent	Passive interaction	Body size	Eating disorder	85	College students	Women paid more attention to the weight-gain areas on the virtual figure than men.
Porras-Garcia et al [96], 2019	Virtual body	Agent	Passive interaction	Body size	Eating disorder	50	Undergraduates	Participants embodied through a synchronous visuotactile technique had higher anxiety-associated body dissatisfaction.
Mertens et al [97], 2019	Virtual body	Agent	Passive interaction	—^c^	Phobias	48	General population and individuals with a fear of spiders	Embodying participants into a virtual body from the 1PP^d^ could improve the validity of fear conditioning.
Provenzano et al [98], 2020	Virtual body	Agent	Passive interaction	Body size	Eating disorder	20	Patients with eating disorders	Different perspectives (1PP vs 3PP^e^) of viewing the virtual body could change people’s body image perception.
Scarpina et al [99], 2019	Virtual body	Agent	Passive interaction	Body size	Eating disorder	30	General population and participants with obesity	A body ownership illusion could be created for both people with obesity and the general population.
Spanlang et al [100], 2018	Virtual body	Agent	Passive interaction	Interaction pattern	Psychosis	27	General population	The body ownership illusion could trigger people’s biological reactions.
Fisher et al [18], 2020	Virtual body	Agent	Passive interaction	Body size	Eating disorder	31	Female adolescents	Body size evaluation results from VR were comparable to paper-based measurements.
Porras-Garcia et al [101], 2020	Virtual body	Agent	Passive interaction	Body size	Eating disorder	41	Female college students	People could have an attentional bias toward the embodied virtual figure because of their own body dissatisfaction.
Gorisse et al [102], 2021	Virtual body	Agent and/or avatar	Explicit interaction	Interaction pattern (engagement level with virtual crowds)	Psychosis	30	General population	Observing a body double successfully engage in a VR social interaction could reduce paranoia.
Wolf et al [103], 2021	Virtual body	Agent and/or avatar	Passive interaction	—	Eating disorder	56	Females with normal weight	Embodiment through a VH could affect people’s self-evaluation of their body weight.
van Gelder et al [104], 2022	Virtual body	Agent and/or avatar	Explicit interaction	Age (current self and future self)	Others	24	Convicted male offenders	Offenders’ interactions with a VH representing their future self could reduce their self-defeating behaviors.
Yamamoto and Nakao [105], 2022	Virtual body	Agent	Passive interaction	Color	Depersonalization	31	Males with various depersonalization tendencies	People with depersonalization had weaker body ownership of the self-representative figure.
Burin et al [106], 2022	Virtual body	Agent and/or avatar	Passive interaction	Embodiment perspectives (1PP or 3PP)	Others	52	General population	Experiencing a moving virtual body from the 1PP could enhance stress coping ability.
Vahle and Tomasik [107], 2022	Virtual body	Avatar	—	Age (age-congruent and age-incongruent with the participants)	Others	74	General population aged between 18 and 30 years	The embodiment of an older avatar increased young adults’ social motivation.
Mendoza-Medialdea et al [108], 2023	Virtual body	Avatar	—	Body size	Eating disorder	57	College women	Manipulating the VH body size could produce changes in body dissatisfaction.
Ascione et al [109], 2023	Virtual body	Avatar	—	—	Eating disorder	23	Adolescent patients with anorexia nervosa	The VR body attentional bias modification task could reduce body-related attentional bias among people with anorexia nervosa.
Schroeder et al [110], 2023	Virtual body	Avatar	—	Body size	Eating disorder	43	Women with high or low body dissatisfaction	People with low body dissatisfaction showed stronger disembodiment during self-avatar mirror exposure.

a VH: virtual human.

b VR: virtual reality.

c Not applicable.

d 1PP: first-person perspective.

e 3PP: third-person perspective.

### Mental Health Conditions

VHs were used in the following research areas in mental health: social anxiety (n=20), eating disorders (n=18), psychosis (n=17), substance abuse (n=5), phobias (n=4), depression (n=3), autism (n=3), PTSD (n=2), dissociative disorders (n=1), and others without specific mental health conditions (n=6).

### Roles of VHs

We categorized the studies based on our taxonomy. Studies with overlapping roles were assigned to their primary role or the one with the highest interactivity. The roles of VHs in the included studies could be categorized into active social interaction partners (n=40), virtual crowds (n=16), and virtual bodies (n=23).

#### Active Social Interaction Partners

In 40 studies, VHs primarily served as active social interaction partners, tested in three different ways: (1) manipulating specific VH characteristics to assess their influence, (2) comparing the responses of clinical and nonclinical individuals, and (3) validating whether VHs in VR could trigger participants’ responses or replicate the effects of real-world experiences.

##### Manipulating VH Characteristics

Twelve studies explicitly examined VH characteristics in social interactions. Wieser et al [53] investigated the anxiety-inducing effects of VH gender and gaze behaviors, finding that male agents with direct gaze elicited stronger anxiety and heart rate elevation among socially anxious women compared to female agents. A similar gender-related effect was observed in a VR fear conditioning program by Reichenberger et al [66], where female patients reported higher anxiety to aversive behaviors (eg, unpleasant air blasts) from male VAs than from female VAs. Park et al [52] and Nijman et al [69] focused on nonverbal behaviors, implementing different facial expressions on VHs to help individuals with psychotic disorders practice emotion recognition.

Researchers also manipulated the dialog responses (positive or negative) [57] and responsive frequency (high or low) [4] in conversation agents. Both the attitude and reply frequency of VHs in a conversation affected participants’ behavioral (eg, interpersonal distance) and physiological (eg, heart rate) responses. In another study by Slater et al [13], participants engaged in a self-conversation by switching between 2 virtual bodies: first embodying a VH resembling themselves to describe their problem to a virtual Sigmund Freud and then embodying Freud to offer advice to their virtual self. This method resulted in a better perception of help compared to scripted dialog. A similar design was then adopted by Yamashita and Yamamoto [79], who found that VR self-counseling with an intimate other avatar was most effective in reducing anxiety. Furthermore, Pan et al [58] explored the impact of VH personality traits (confident vs shy) on participants’ perceptions, showing that the shy VH was perceived as more positive and friendly. The effects of VH emotional attributes were further demonstrated by Wei et al [37], who showed that a virtual coach with positive facial expressions and affirmative nods enhanced participants’ therapeutic alliance and treatment confidence during a VR fear-of-heights consultation. Lastly, the perceived agency of VHs was studied by Kothgassner et al [45,64], who compared participants’ reactions when they believed they were interacting with a human versus a computer. Both experiments indicated that interactions with VHs can be as effective as interactions with real-life counterparts, provided participants believe the VH is controlled by a human in real time.

##### Comparing Clinical and Nonclinical Populations

In another use case, 5 studies used active interaction partners to assess the differences between clinical and nonclinical populations. Han et al [56] found that patients with schizophrenia avoided eye contact more often when interacting with 2 VHs in a 3-person conversation. Lee et al [71] used a ball game to study self-disturbances (eg, a distorted perception of one’s own body boundary), suggesting that patients with schizophrenia showed longer reaction times and a weaker sense of body boundary compared to controls. Robitaille et al [60] designed a scenario to detect executive functioning difficulties in military personnel with traumatic brain injury. In a military patrol scene, a virtual male dressed as a civilian was used to simulate a divided attention task by approaching the participant. Both groups tolerated the experience, but those with posttraumatic brain injury displayed navigation difficulties after a divided attention task was added. Lastly, Fusaro et al [7] and Artiran et al [76] studied the differences in responses to VHs between participants with autism and nonclinical controls, and found that individuals with autism maintained greater interpersonal distance and responded less to VHs’ social cues (eg, head turning and direct gaze) during virtual interviews.

##### Validation Studies With VHs

Twenty-two studies used active interaction partners to understand whether VHs in VR could trigger participants’ responses or replicate the effects of a real-world experience. Ten studies involved participants with mental health conditions or specific phobias. Three of them were related to psychosis. Percie du Sert et al [61] tested a therapy for auditory verbal hallucinations in psychosis. Patients interacted with a VH representing the harmful voice they heard, and this exposure reduced their auditory verbal hallucination–related depressive symptoms. Freeman et al [9] developed gameChange, an automated VR therapy designed to address agoraphobia in psychosis. Patients were guided by a virtual coach through everyday scenarios like visiting a general practitioner or shopping, resulting in significant anxiety reduction, particularly in those with severe symptoms. More recently, Freeman et al [10] launched Phoenix, where a coach named Farah helps young patients with psychosis build self-confidence through guided tasks. Two other studies focused on eating disorders. Bektas et al [74] used a virtual kitchen scene in patients with anorexia nervosa, finding that a VH encouraging patients to challenge their disorder elicited stronger disgust reactions toward food. Natali et al [77] used the same scenario to induce positive mood, showing that supportive VHs can reduce anxiety and improve mood in patients with anorexia. Regarding phobias, Freeman et al [62] conducted a clinical trial for treating fear of heights. A VR coach called Nic guided participants through the different stages of the experience, leading to a significant reduction in their fear. Similarly, Miloff et al [68] used a holographic coach for spider phobia, where positive relationships predicted better outcomes. Falconer et al [11] promoted self-compassion in depression through role-swapping between a compassionate adult and a virtual child. Amaral et al [6] conducted a clinical trial using VHs for social and cognitive training in individuals with autism. A further study [75] applied VR to treat addiction to cannabis. Participants interacted with a VH representing a key figure in their cannabis consumption, such as a friend, a drug dealer, or those triggering their cravings, showing potential to reduce usage.

Among the remaining 13 validation studies involving nonclinical populations, 6 focused on social anxiety. Kwon et al [54] compared the effects of displaying VHs on a desktop monitor versus an immersive VR system, finding that immersive VR elicited stronger anxiety. This was further supported by a VR self-compassion induction [59] and a joint attention study [67], both of which concluded that VR had a greater capacity to induce anxiety compared to nonimmersive setups. Powers et al [55] examined participants’ fear levels during VR and real-life interactions, noting that VR interactions triggered fear and anxiety similar to real-life situations. Banakou et al [80] suggested that single-session VR exposure could match multisession efficacy for public speaking anxiety. Moreover, Quintana et al [65] investigated the effects of smells on the perceptions of VHs. They discovered that exposure to fear-related odorants increased anxiety and reduced trust toward the virtual character. Other applications included improving depression via compassion practice [12], testing a customizable VH-based auditory hallucination therapy tool with hospital staff [72], and simulating alcohol refusal scenarios with VHs in VR FestLab [73]. Similar refusal training targeted gaming addiction [63] and nicotine addiction [51]. Finally, VR was used to promote well-being and self-confidence in youth by fostering strength awareness [70] and reducing self-criticism [78] through structured conversations with virtual characters.

### Virtual Crowds

Sixteen studies used virtual crowds. Six of them were related to anxiety. In 1 of the earliest studies, Slater et al [3] compared public speaking to an attentive versus a disruptive virtual audience, finding that crowds could evoke anxiety comparable to real life, with better performance when the audience was attentive. Similar findings were reported by Takac et al [87], who suggested that the distress created in a virtual public speaking scenario could have lingering effects after the VR session. Virtual crowds were also used to populate a virtual office, finding that watching other VHs working was effective in triggering work-related stress [49] or creating peer effects to make people work more efficiently [14]. Brinkman et al [48] explored the effect of the size and ethnicity of virtual crowds in a bar scene, showing that VR exposure to an increased density and proportion of VHs with other ethnicities could induce stronger distress. Additionally, Shiban et al [84] incorporated air blasts and the sound of a female character screaming when participants approached a virtual crowd to study how individuals develop social fears and how these fears can be diminished or overcome through repeated exposure.

Six studies used a virtual crowd for research on psychosis. Early tests examined people’s appraisals of neutral characters in a library scene, demonstrating that virtual crowds elicited persecutory thoughts for insights into psychosis [81,82]. Later research employed daily VR scenarios populated with VHs, such as in an underground train or school canteen, to understand factors associated with paranoid thinking. The findings suggested that paranoia could be predicted by anxiety, worry, perceptual anomalies, and cognitive inflexibility [15], and was influenced by individuals’ self-confidence [86]. For adolescents, interpersonal threat or hostility heightened state paranoia [89]. Further, Wei et al [88,111] found that VH facial design influenced both paranoia and visual attention.

Mountford et al [85] used a virtual crowd in a virtual bus ride to study eating disorders. Participants were tested for their body image satisfaction, and the results showed that exposure to the virtual scenario with VHs did not necessarily change people’s current feelings or perceptions about their body image. Cho et al [83] designed a VR bar to study alcohol cravings and found that the introduction of drinking characters led to a stronger drinking desire compared to simply displaying the alcohol in the scene. Similarly, Rovira et al [90] found that VR exposure to smoking-cue VHs elicited stronger cigarette cravings than neutral environments without VHs. Rizzo et al [39] used virtual soldiers and civilians for military training and found a reduction in PTSD severity in 80% of the study completers.

### Virtual Bodies

VHs were used as body representations in 20 studies, with 15 focusing on eating disorders. Riva and Melis [1] conducted the first study in the literature on body image representation using VR. Participants selected virtual figures representing their current and ideal body size. Perpiñá et al [91] introduced a feature allowing patients with eating disorders to adjust the size of a virtual body to reflect their body image. Both showed VR’s feasibility for assessing body image disturbances, with results comparable to paper-based measures but showing greater engagement [18]. While these early studies did not induce body ownership, later studies often aimed to do so.

Studies employing the body ownership illusion paradigm found that embodying a larger virtual figure led participants to check their virtual body more frequently [94,95] and experience a higher degree of self-dissatisfaction and anxiety [40,96,98,101,108]. In contrast, Schroeder et al [110] found that body dissatisfaction was not necessarily affected by the weight of the avatar, although people with higher body dissatisfaction exhibited more body-checking behaviors in weight-related areas.

Ferrer-Garcia et al [93] created a paradigm to reduce anxiety related to body image in female students by making them return to the normal-size virtual figure after experiencing a larger body. A similar method was used to alleviate body image distortion in both healthy-weight participants and those with obesity [99]. Wolf et al [103] compared the body weight perceptions of 2 groups of females: one that embodied a VH and another that observed the VH as another person’s avatar. The self-embodiment group underestimated the body weight of the VH compared to those who rated the VH as another’s avatar. Furthermore, Ascione et al [109] conducted a body-related attention bias modification task to reduce excessive visual focus on the weight-related areas of a self-represented VR body, which significantly decreased body dissatisfaction.

Three other studies used virtual bodies for psychosis research. Spanlang et al [100] examined whether self-fragmentation in VR, where participants embodied a VH while retaining physical presence in the real world, impacted the biological responses in patients with schizophrenia. Yamamoto and Nakao [105] found that perceiving a fake body as one’s own reduced body ownership in depersonalization. Gorisse et al [102] used a virtual body double resembling each participant for a social VR task. They concluded that observing a body double engaging in VR social interactions could reduce persecutory thoughts during the task.

Additionally, van Gelder et al [104] demonstrated that alternating convicted offenders between their current and aged future selves reduced self-defeating behaviors. Similarly, embodying an older body could increase social motivation among young adults [107]. Besides, Burin et al [106] presented participants with a moving character from first-person and third-person perspectives. They concluded that a body illusion from the first-person perspective created stronger physiological activation to help people practice stress coping. Aymerich-Franch et al [92] assessed the impact of the facial similarity of a VH on participants, finding that embodying a figure with a dissimilar face reduced anxiety during VR presentations.

### VAs and Avatars

Out of the 79 studies, 74 involved the use of VAs. Nine of them adopted a semiautonomous agent design, where facilitators manually triggered an audio effect [3,61], adjusted VH voices [75], or played prerecorded animations [4,55] to customize characters’ behaviors in real time.

Regarding the interaction types of VAs, approximately half (39/74, 53%) engaged in explicit interactions with participants, primarily serving as active interaction partners. In 10 studies, VAs exhibited implicit interactions (eg, occasionally looking at participants and then looking away). Passive interactions were observed in 26 studies where VHs were used as virtual crowds and virtual bodies.

In the reviewed studies, the term “avatar” was often used to describe any type of VH, even when they did not represent the participant’s self. Only 5 studies used VHs as participant avatars. For example, Aymerich-Franch et al [92] used an avatar with varying levels of visual similarity to the participant’s face to study the self-embodiment experience. Additionally, 12 studies combined the use of agents and avatars to create perspective changes through body-swapping experiences in social interactions [11,13,104] or to explore the effects of vicarious agency (the perception of control over the VH) [102]. Kothgassner et al [45,64] further examined the effect of perceived agency by comparing participants’ VR experiences with VAs and avatars. The results indicated that avatars could elicit stronger emotional responses compared to VAs.

### Human Characteristics

Of the 79 studies reviewed, 44 examined the effects of manipulating specific characteristics of VHs. These manipulations aimed to assess how physical and behavioral attributes of VHs influence participants’ perceptions and behavioral responses. The manipulated features included their visual appearance [48,52,99] (eg, gender, ethnicity, and body size), interaction behavior [7,76,87] (eg, interpersonal distance, eye gaze behavior patterns, and responsiveness toward participants), perceived agency [45,64] (VA or avatar), and emotions [52,75] (eg, emotional facial expressions) or personality [58]. While most of the traits were nonverbal behaviors, 4 studies explored the effects of verbal behaviors, including dialog content [13], conversation attitude (eg, positive vs neutral dialog) [56,57], and the voice of VHs [75]. The most frequently studied trait was body size (n=13), while traits, such as sense of affirmation [37] and personality [58], received limited investigation.

In addition to individual traits, 2 studies examined the effects of crowd size and density as independent variables in virtual crowd settings [48,87]. These studies explored how variations in the number and arrangement of virtual characters influence participants’ sense of presence and anxiety in crowded environments.

## Discussion

### Summary of Findings

This paper reviewed VR studies that included VHs in scenarios to understand and treat mental health difficulties. We examined 79 studies and categorized them based on the primary role of the VHs: active social interaction partners, virtual crowds, and virtual bodies. We focused on the use of agency, types of interactions, and characteristics of the VHs. VHs have served diverse purposes and demonstrated the ability to create psychological, behavioral, and physiological influences. However, few studies have provided comprehensive descriptions of VHs’ visual appearance or behavioral capabilities. Without such details, it becomes challenging to assess their actual impact, and replicability is reduced. The potential of each VH role requires further exploration to address these gaps and the associated challenges.

### Active Social Interaction Partners

Active social interaction partners interact explicitly with participants. A persistent challenge in this context has been enhancing the realism and responsiveness of these interactions, particularly during close-up engagements [19,112,113]. Improving both visual fidelity and behavioral plausibility is essential for achieving lifelike interactions [30,114,115]. Consistent design of both visual and behavioral elements is needed to avoid the uncanny valley, where characters appear almost human but evoke discomfort due to subtle imperfections, as also seen in VR interactions [116,117]. Additionally, it is important to consider VH behavioral plausibility and consistency across multiple behavioral channels, including both verbal and nonverbal communication (eg, body movement, facial expressions, voice, and dialog content). For example, Choudhary et al [118] demonstrated that mismatches between facial expressions and vocal tone (eg, a happy face with an unhappy voice) negatively impacted participants’ immersion and engagement, highlighting the importance of aligning these communication modes.

Regarding responsiveness and interactivity, current feedback in mental health VR often relies on prerecorded actions triggered by participant inputs, such as user interface selection, position, or response time [49,60,65]. Future applications could integrate machine learning algorithms, such as reinforcement learning, to create autonomous VHs that learn from experience [119]. These VHs could adjust parameters, such as the interpersonal distance, to maximize attention and keep participants engaged. Algorithms could detect interaction dynamics (eg, participant attitudes and responsiveness) and adjust VH behaviors in real-time to achieve specific therapeutic goals, particularly in applications encouraging positive behaviors. Another area to explore is the real-time monitoring of physiological responses to create adaptive interactions based on participants’ affective state. A few of the studies reviewed used biometric data (eg, salivary alpha-amylase, heart rate, and galvanic skin) to evaluate physiological responses [52,100,106], but none leveraged these data to enhance interaction fidelity. Real-time estimates of emotional arousal and valence could enable VHs to respond meaningfully to participants’ emotional changes [120].

A virtual coach represents a specific type of active social interaction partner, designed to guide and motivate users toward positive behavioral changes while facilitating the automation of therapy delivery. Focus group feedback indicated that users valued the convenience, accessibility, and anonymity provided by a virtual coach, but they also expressed concerns about the coach feeling unrelatable and difficult to engage with [121]. The development of a VR coach should incorporate knowledge and feedback from domain experts and targeted user groups to ensure it is tailored to the specific age range and mental health conditions it aims to address [122,123]. Generally, increasing the engagement and friendliness of communication from virtual coaches is likely to improve their effectiveness and the outcomes of VR therapy [68].

### Virtual Crowds

In virtual crowd studies, it is standard practice to use identical designs and behaviors for characters that share common objectives to achieve a balance between realism and computational efficiency [124,125]. However, visual and behavioral repetition patterns in a group of VHs are very noticeable and decrease fidelity. Introducing variations in appearance and movement is important to diversify crowds and avoid these patterns [126,127]. Visual diversity can be achieved, for example, by randomizing height, body shape, hair color, and clothing. Behavioral diversity can be enhanced by blending animations, using motion capture data from multiple individuals, and implementing procedural animations with a certain degree of randomization to generate varied movements in real-time based on environmental and interaction dynamics.

There are additional considerations. When virtual crowds have autonomy to move around a space, each one should navigate naturally, avoiding collisions with static and dynamic obstacles. For example, Trivedi and Mousas [128] implemented a crowd behavior system for a VR street scene, where each VA had navigation scripts with steering and pathfinding capabilities to avoid collisions. Each agent also had a customizable target location, walking speed, and animation (eg, head direction and gaze pattern). Other advanced virtual crowd control systems generate real-time behaviors driven by user input and the dynamics of other virtual entities within the crowd [129]. These designs could be applied in mental health applications to improve crowd interactions and better understand responses to busy environments.

### Virtual Bodies

Studies involving virtual bodies predominantly helped individuals reassess their body image biases or gain new perspectives, provided by the body ownership illusion. These use cases require improved methods to enable the sense of embodiment. Existing methods like visual-motor synchrony [97] and visual-tactile synchrony [96,99] are designed to align visual feedback with participants’ movements or tactile sensations to enhance the illusion of ownership over the virtual body. While effective in initiating this illusion, sustaining or manipulating it throughout the VR experience remains challenging. Mismatched sensory feedback or slight discrepancies in motion can disrupt the sense of embodiment [130,131]. Integrating other sensory modalities, such as auditory feedback (eg, footsteps and voice modulation) or olfactory feedback (eg, simulating a scent experience based on the participant’s location), could enhance the body ownership illusion. Combined multisensory feedback has been shown to strengthen and prolong the influence on embodiment, affect, and behaviors [132,133].

Customizing virtual bodies to closely resemble participants can enhance the sense of embodiment. Aymerich-Franch et al [92] found that embodying a virtual body with a similar face increased self-association and anxiety during public speaking tasks compared to dissimilar faces. This provides evidence for the importance of avatar personalization and its potential to modulate emotional responses. Customization should consider not only physical appearance but also factors like ethnic background [134] and voice [135]. These factors can significantly contribute to the body ownership illusion.

### Ethical Considerations and Recommendations

The integration of VHs in mental health VR raises potential ethical and methodological challenges. A key concern is the lack of standardization in the technical implementation of character creation and animation. Many implementations rely on proprietary or nonstandardized software, which complicates replication and cross-study comparison. When such tools become inaccessible or difficult to adopt, scaling the experiments or building cumulative evidence becomes challenging. Moving toward open-source platforms or developing shared technical standards would enhance reproducibility and research efficiency. Encouragingly, the computer science community has started to introduce open-source 3D character libraries [26-28], some of which have already been used in mental health research [11,13]. This provides early examples of community-driven resources that could serve as a foundation for broader standardization.

Equally important is the transparency of reporting with regard to VHs. Many current studies provide limited descriptions of VH characteristics, and some even omit a general description of VR scenes. Such omissions reduce study transparency and limit replicability for continuous research. General ethical guidelines from the UK National Institute for Health and Care Excellence (NICE) [136] emphasize clear reporting of system design and capabilities for therapeutic applications. The CONSORT-EHEALTH extension recommends that digital health interventions specify intervention components, delivery modes, tailoring, and technical features [137]. More specifically, RATE-XR (reporting for the early-phase clinical evaluation of applications using extended reality) provides a reporting framework for early-phase clinical extended reality applications, with a checklist covering application characteristics, hardware, software, and development details [138]. For more tailored standards, VH-specific reporting practices, ideally supported by visual and technical documentation (eg, screenshots, videos, and model specifications), are needed to improve reproducibility and comparability across disciplines.

Moreover, data privacy and participant safeguards also require careful consideration. VH-mediated applications can sometimes collect sensitive physiological, behavioral, and conversational data [13,48,139], raising risks related to identity inference and emotional profiling, where user behavior data can sometimes be used to infer sensitive personal traits [140]. In addition, embodiment-based interactions can sometimes provoke unintended psychological effects, such as distress or overidentification with the VH [141,142]. To mitigate these risks, researchers should implement robust informed consent procedures and ensure secure data handling. Safeguards, such as participant prescreening, clinician oversight, built-in safety controls (eg, pause or exit functions in VR software), and structured debriefing protocols, are essential to protect participants.

### Limitations

This systematic review has several limitations. First, our search strategy may not have captured all relevant VR mental health studies involving VHs. We used 5 databases and conducted searches limited to titles and abstracts with fixed keyword combinations, excluding studies that did not simultaneously mention VR, VHs, and mental health. To maintain a consistent search strategy across platforms, we did not use controlled vocabulary, such as Medical Subject Headings (MeSH) in PubMed, which may have further reduced recall. To mitigate this, we conducted citation chaining and identified 6 additional eligible studies not captured in the original search. However, the omission may have still remained, and it may have narrowed the evidence base. Future updates should refine the search strategy by incorporating broader synonyms and controlled vocabulary, and including gray literature sources.

Second, there are limitations in our categorization of VH roles and characteristics. Existing taxonomies used to classify virtual characters and their behaviors [30,143] are at least a decade old and do not specifically focus on VR studies in mental health. We developed our own classification based on the usage of VHs in the reviewed studies. This framework is tailored to mental health research, and it may require adaptation for application in other research areas. Ongoing efforts to develop and validate updated, domain-general taxonomies would improve consistency and comparability across studies.

Third, most included studies were pilot, feasibility, or exploratory in nature, often with small sample sizes and limited follow-up. While we included only empirical studies with more than one participant to enhance rigor, the predominance of early-stage designs may have constrained overall study quality and generalizability. Future research could prioritize more robust designs, such as randomized controlled trials or longitudinal studies with quality assessments, to generate stronger and more interpretable evidence.

Fourth, regarding result analysis, we opted for a narrative review approach instead of a quantitative evaluation to measure the quality and effectiveness of VHs across studies. This choice was made due to the variability in study objectives (ranging from validation and assessment to clinical treatments) and the diversity in participant characteristics. In addition, many studies lacked sufficient descriptions of VH attributes and consistent reporting on VR systems and software, which constrained systematic comparison and aggregation. As the field progresses and reporting becomes more standardized, meta-analyses and formal quality appraisals will become feasible.

### Conclusions

VHs can be used in many different ways within immersive VR mental health research and have served primarily as active interaction partners, virtual crowds, and virtual bodies. Across the 79 studies reviewed, most VHs were computer-controlled agents, with perceived agency shaping emotional and behavioral responses. Research to date has predominantly examined body size and gender, with limited attention to emotional expression or personality traits. Many studies provided insufficient technical and visual details on VH design, limiting reproducibility and cross-study comparison. Addressing these gaps is crucial for advancing the evidence base and understanding how specific VH characteristics influence mental health outcomes in VR. To our knowledge, this review provides the first synthesis of the use of VHs in VR mental health, offering a potential foundation for future VR programming of VHs and future studies on their implementation.

## Supplementary material

10.2196/75087 Multimedia Appendix 1 Search query details.

10.2196/75087 Checklist 1 PRISMA checklist.

## Abbreviations

PRISMA: Preferred Reporting Items for Systematic Reviews and Meta-Analyses
PTSD: posttraumatic stress disorder
VA: virtual agent
VH: virtual human
VR: virtual reality
