# Supplementary material for: Virtual Humans in Virtual Reality Mental Health Research: Systematic Review
Source: JMIR XR Spat Comput. 2025 Oct 8;2:e75087. doi: 10.2196/75087 (PMC12671307; doi:10.2196/75087)
Supplement: Multimedia Appendix 1 [file xr-v2-e75087-s001.docx]

**Appendix 1 – Search Details**

| Database | Platform | Number of results (Nov 24, 2025) |
| --- | --- | --- |
| MEDLINE | Ovid | 172 |
| PubMed | - | 233 |
| APA PsycInfo | Ovid | 247 |
| Scopus | Elsevier | 1232 |
| Web of Science | Clarivate | 712 |
| *Total* |  | 2596 |

*Keyword search restricted to Title, Abstract; search conducted on November 20, 2024.

**PubMed**

( (virtual reality[Title/Abstract] OR immersive virtual reality[Title/Abstract] OR VR[Title/Abstract]) AND (virtual human[Title/Abstract] OR virtual character[Title/Abstract] OR virtual agent[Title/Abstract] OR avatar[Title/Abstract] OR humanoid[Title/Abstract]) AND ( (assessment[Title/Abstract] OR treatment[Title/Abstract] OR therapy[Title/Abstract] OR "mental health"[Title/Abstract]) OR ("mood disorders"[Title/Abstract] OR depress*[Title/Abstract] OR bipolar[Title/Abstract] OR mania[Title/Abstract] OR paranoia[Title/Abstract] OR psychosis[Title/Abstract] OR psychotic[Title/Abstract] OR schizophren*[Title/Abstract] OR schizotyp*[Title/Abstract] OR delus*[Title/Abstract] OR hallucinat*[Title/Abstract] OR phobias[Title/Abstract] OR "obsessive compulsive disorder"[Title/Abstract] OR OCD[Title/Abstract] OR anxiety[Title/Abstract] OR "post traumatic stress disorder"[Title/Abstract] OR PTSD[Title/Abstract] OR trauma[Title/Abstract] OR "anorexia nervosa"[Title/Abstract] OR "bulimia nervosa"[Title/Abstract] OR "eating disorders"[Title/Abstract] OR "binge eating"[Title/Abstract] OR insomnia[Title/Abstract] OR sleep[Title/Abstract] OR nightmares[Title/Abstract] OR circadian[Title/Abstract] OR panic[Title/Abstract] OR substance[Title/Abstract] OR abuse[Title/Abstract] OR cannabis[Title/Abstract] OR tobacco[Title/Abstract] OR alcohol[Title/Abstract] OR amphetamine[Title/Abstract] OR hallucinogens[Title/Abstract] OR heroin[Title/Abstract]) ) )

**MEDLINE**

((virtual reality or immersive virtual reality or VR).ti,ab. AND

(virtual human or virtual character or virtual agent or avatar or humanoid).ti,ab. AND

((assessment or treatment or therapy or "mental health").ti,ab. OR

("mood disorders" or depress* or bipolar or mania or paranoia or psychosis or psychotic or schizophren* or schizotyp* or delus* or hallucinat* or phobias or "obsessive compulsive disorder" or OCD or anxiety or "post traumatic stress disorder" or PTSD or trauma or "anorexia nervosa" or "bulimia nervosa" or "eating disorders" or "binge eating" or insomnia or sleep or nightmares or circadian or panic or substance or abuse or cannabis or tobacco or alcohol or amphetamine or hallucinogens or heroin).ti,ab.))

**APA PsycInfo**

(("virtual reality" or "immersive virtual reality" or VR).ti,ab,id.

or exp Virtual Reality/)

AND

(("virtual human" or "virtual character" or "virtual agent" or avatar or humanoid).ti,ab,id.

or exp Avatars/)

AND

((assessment or treatment or therapy or "mental health").ti,ab,id.

or exp Mental Health/

or "mood disorders".ti,ab,id. or exp Mood Disorders/

or depress*.ti,ab,id. or exp Depression/

or bipolar.ti,ab,id. or exp Bipolar Disorders/

or mania.ti,ab,id. or exp Mania/

or paranoia.ti,ab,id. or exp Paranoia/

or psychosis.ti,ab,id. or psychotic.ti,ab,id. or exp Psychosis/

or schizophren*.ti,ab,id. or exp Schizophrenia/

or schizotyp*.ti,ab,id. or exp Schizotypal Personality Disorder/

or delus*.ti,ab,id. or exp Delusions/

or hallucinat*.ti,ab,id. or exp Hallucinations/

or phobias.ti,ab,id. or exp Phobias/

or "obsessive compulsive disorder".ti,ab,id. or OCD.ti,ab,id. or exp Obsessive Compulsive Disorder/

or anxiety.ti,ab,id. or exp Anxiety Disorders/

or "post traumatic stress disorder".ti,ab,id. or PTSD.ti,ab,id. or exp Posttraumatic Stress Disorder/

or trauma.ti,ab,id. or exp Psychological Trauma/

or "anorexia nervosa".ti,ab,id. or exp Anorexia Nervosa/

or "bulimia nervosa".ti,ab,id. or exp Bulimia Nervosa/

or "eating disorders".ti,ab,id. or exp Eating Disorders/

or "binge eating".ti,ab,id. or exp Binge Eating Disorder/

or insomnia.ti,ab,id. or exp Insomnia/

or sleep.ti,ab,id. or exp Sleep/

or nightmares.ti,ab,id. or exp Nightmares/

or circadian.ti,ab,id. or exp Circadian Rhythms/

or panic.ti,ab,id. or exp Panic Disorder/

or substance.ti,ab,id. or exp Substance Abuse/

or abuse.ti,ab,id. or exp Drug Abuse/

or cannabis.ti,ab,id. or exp Marijuana/

or tobacco.ti,ab,id. or exp Tobacco Smoking/

or alcohol.ti,ab,id. or exp Alcohol Drinking Patterns/

or amphetamine.ti,ab,id. or exp Amphetamines/

or hallucinogens.ti,ab,id. or exp Hallucinogens/

or heroin.ti,ab,id. or exp Heroin/)

**Web of Science Core Collection**

TS=(("virtual reality" OR "immersive virtual reality" OR "VR") AND

("virtual human" OR "virtual character" OR "virtual agent" OR avatar OR humanoid) AND

((assessment OR treatment OR therapy OR "mental health") OR

("mood disorders" OR depress* OR bipolar OR mania OR paranoia OR psychosis OR psychotic OR schizophren* OR schizotyp* OR delus* OR hallucinat* OR phobias OR "obsessive compulsive disorder" OR OCD OR anxiety OR "post traumatic stress disorder" OR PTSD OR trauma OR "anorexia nervosa" OR "bulimia nervosa" OR "eating disorders" OR "binge eating" OR insomnia OR sleep OR nightmares OR circadian OR panic OR substance OR abuse OR cannabis OR tobacco OR alcohol OR amphetamine OR hallucinogens OR heroin)))

**Scopus**

TITLE-ABS-KEY ( ( "virtual reality" OR "immersive virtual reality" OR VR ) AND ( "virtual human" OR "virtual character" OR "virtual agent" OR avatar OR humanoid ) AND ( assessment OR treatment OR therapy OR "mental health" OR "mood disorders" OR depress* OR bipolar OR mania OR paranoia OR psychosis OR psychotic OR schizophren* OR schizotyp* OR delus* OR hallucinat* OR phobia* OR "obsessive compulsive disorder" OR OCD OR anxiety OR "post traumatic stress disorder" OR PTSD OR trauma OR "anorexia nervosa" OR "bulimia nervosa" OR "eating disorder*" OR "binge eating" OR insomnia OR sleep OR nightmare* OR circadian OR panic OR substance OR abuse OR cannabis OR tobacco OR alcohol OR amphetamine* OR hallucinogen* OR heroin ) ) AND ( LIMIT-TO ( LANGUAGE , "English" ) ) AND ( LIMIT-TO ( DOCTYPE , "ar" ) OR LIMIT-TO ( DOCTYPE , "cp" ) )
